# Adequately Diversified Dietary Intake and Iron and Folic Acid Supplementation during Pregnancy Is Associated with Reduced Occurrence of Symptoms Suggestive of Pre-Eclampsia or Eclampsia in Indian Women

**DOI:** 10.1371/journal.pone.0119120

**Published:** 2015-03-18

**Authors:** Sutapa Agrawal, Jasmine Fledderjohann, Sukumar Vellakkal, David Stuckler

**Affiliations:** 1 South Asia Network for Chronic Disease, Public Health Foundation of India, Gurgaon, Haryana, 122022, India; 2 Department of Sociology, Oxford University, Oxford, United Kingdom; University of Ottawa, CANADA

## Abstract

**Background/Objective:**

Pre-eclampsia or Eclampsia (PE or E) accounts for 25% of cases of maternal mortality worldwide. There is some evidence of a link to dietary factors, but few studies have explored this association in developing countries, where the majority of the burden falls. We examined the association between adequately diversified dietary intake, iron and folic acid supplementation during pregnancy and symptoms suggestive of PE or E in Indian women.

**Methods:**

Cross-sectional data from India’s third National Family Health Survey (NFHS-3, 2005-06) was used for this study. Self-reported symptoms suggestive of PE or E during pregnancy were obtained from 39,657 women aged 15-49 years who had had a live birth in the five years preceding the survey. Multivariable logistic regression analysis was used to estimate the association between adequately diversified dietary intake, iron and folic acid supplementation during pregnancy and symptoms suggestive of PE or E after adjusting for maternal, health and lifestyle factors, and socio-demographic characteristics of the mother.

**Results:**

In their most recent pregnancy, 1.2% (n=456) of the study sample experienced symptoms suggestive of PE or E. Mothers who consumed an adequately diversified diet were 34% less likely (OR: 0.66; 95% CI: 0.51-0.87) to report PE or E symptoms than mothers with inadequately diversified dietary intake. The likelihood of reporting PE or E symptoms was also 36% lower (OR: 0.64; 95% CI: 0.47-0.88) among those mothers who consumed iron and folic acid supplementation for at least 90 days during their last pregnancy. As a sensitivity analysis, we stratified our models sequentially by education, wealth, antenatal care visits, birth interval, and parity. Our results remained largely unchanged: both adequately diversified dietary intake and iron and folic acid supplementation during pregnancy were associated with a reduced occurrence of PE or E symptoms.

**Conclusion:**

Having a adequately diversified dietary intake and iron and folic acid supplementation in pregnancy was associated with a reduced occurrence of symptoms suggestive of PE or E in Indian women.

## Introduction

Pre-eclampsia is a pregnancy-induced hypertensive disorder characterized by high blood pressure and proteinuria, i.e. elevated levels of protein in the urine (used to distinguish pre-eclampsia from gestational hypertension), after the 20^th^ week of pregnancy [1]. Eclampsia is defined as the occurrence of generalized seizures/convulsions and/or unexplained coma during pregnancy or the postpartum period in the absence of other neurologic conditions such as epilepsy [2–3]. Pre-eclampsia or eclampsia (PE or E) is one of the leading causes of maternal and fetal mortality and morbidity worldwide [4], and is associated with adverse pregnancy outcomes including perinatal death, preterm birth, and intrauterine growth retardation [1,5]. Based largely on clinical data, the incidence of pre-eclampsia is between 2 and 10%, depending on the population studied and definition of pre-eclampsia used [6]; clinical studies suggest that the proportion of deliveries impacted by PE or E in Indian women ranges from as low as 0.9% to as high as 7.7% of all deliveries [7–9]. However, these clinical studies are likely to suffer from selection bias on the basis of severity of the condition, especially among populations with limited access to prenatal care, and therefore may underestimate the prevalence of the condition. Precise country-specific population level estimates of PE or E prevalence are largely unavailable.

Although the etiology of PE or E remains unclear, the role of maternal diet in the development of PE or E has recently received increased attention [10–11]. Several studies indicate that micronutrient deficiencies, such as magnesium, vitamins A and C, folic acid [12], and calcium [13–15] may contribute to PE or E risk. The evidence of a beneficial effect of maternal iron supplementation during pregnancy is particularly compelling [16]. As well, three large-scale cohort studies of the association between folic acid supplements containing multivitamins and gestational hypertension, including PE [12, 15,17–18], all show a protective effect of folic acid supplementation on pre-eclampsia. A recent large cohort study from Denmark [19] shows that regular use of folic acid in pregnancy is related to a reduced risk of pre-eclampsia among normal-weight women. However, two recent studies in China [20] and Holland [21] fail to find an effect of folic acid supplementation on pre-eclampsia or gestational hypertension. The links between micronutrient supplementation and PE or E have largely been assessed in clinical trials, but there is an urgent need for research examining links between PE or E and adequately diversified dietary intake during pregnancy more broadly, especially in light of research suggesting that nutrients may be better-absorbed from food sources than from supplements [22].

Several epidemiologic studies indicate that consumption of fruits, vegetables, and dietary fibre is associated with lower pre-eclampsia risk [23–26], possibly influencing pre-eclampsia through intestinal anti-inflammatory mechanisms [27]. It is hypothesized that inadequate antioxidant and folate intake may contribute to oxidative stress, thereby increasing the risk of pre-eclampsia [28]. Dietary intake, then, may shape PE or E risk by influencing micronutrient and antioxidant levels. For example, lycopene, an antioxidant found in many red fruits and vegetables, has been associated with a reduced risk of PE or E [29]. Dietary diversity—i.e. regular consumption of food items across a broad range of food groups—is widely recognized as a key dimension of diet quality, reflecting access to a variety of foods and serving as a proxy for individual nutrient adequacy [30]. There is ample evidence from developed countries showing that dietary diversity is indeed strongly associated with nutrient adequacy. Unfortunately there is a dearth of studies from developing countries, but the few available studies support an association between dietary diversity and nutrient adequacy [31–33].

Associations between diversified dietary intake, micronutrients, and hypertensive disorders are particularly relevant in India, where rates of malnutrition and micronutrient deficiencies are high among pregnant women [34]. To our knowledge, there has not been any previous large-scale population based study of the dietary risk factors for PE or E in Indian women. Studies which have examined the links between diet and PE or E empirically have not been conducted in high burden countries; nor have they employed appropriate multivariable models. Similarly, much research has focused on treatment of PE or E, but less is known about potential preventive factors, particularly those associated with maternal behaviours during pregnancy [35–36].

Identifying the link between an adequately diversified dietary intake and the risk of PE or E may suggest an important point of intervention at both the peri-conceptional and gestational stages particularly in a developing country such as India. In this study, as dietary diversity is a key factor in ensuring an adequate micronutrient balance, we use the large-scale, cross-sectional and nationally representative third National Family and Health Survey (NFHS-3) data [37–38] to test the hypothesis that adequately diversified dietary intake and iron and folic acid supplementation during pregnancy are inversely related to the risk of PE or E among Indian women.

## Methodology

Cross-sectional data from the most recent wave of the National Family Health Survey (NFHS-3, 2005–2006), India’s Demographic and Health Survey (DHS), was used for this study. These data are publicly available by request from DHS [37]. The survey was approved by the ethics boards of the implementing agencies in the respective states of India and by the Indian government [38]. Our analysis used secondary survey data which was completely anonymised prior to access. As the data were anonymised by the DHS [37], no ethics board review was required. Using a multistage randomised cluster design (response rate = 98%), NFHS-3 employed an interviewer-administered questionnaire in the native language of the respondent to collect sociodemographic and health information, resulting in a nationally representative probability sample of 124,385 women aged 15–49 years. All states of India are represented in the sample (except the Union Territories covering less than 1% of India’s population). Full details of the survey have been published elsewhere [38].

To assess symptoms of PE or E, we restricted the sample to those women who had a live birth in the five years preceding the survey. We further restricted our analyses to data pertaining to the most recent birth, both to minimize recall bias and in order to draw on iron and folic acid supplementation and antenatal care (ANC) measures, which were only available for only the most recent pregnancy. This resulted in a final sample size of 39,657 participants.

### Outcome Measure

To assess the occurrence of PE or E, we constructed a measure based on women’s self-reports of symptoms during pregnancy. Specifically, mothers were asked: “During this pregnancy, did you have difficulty with your vision during daylight?”, “During this pregnancy, did you have swelling of the legs, body or face?”, and “During this pregnancy, did you have convulsions not from fever?” The response options were “Yes”, “No”, and “Don’t know”. Following the World Health Organisations [39] and National Institute for Health and Care Excellence guidelines [40], we created a dichotomous indicator of PE or E: Women who reported both difficulty with vision during daylight and swelling of the legs, body, or face, were coded as having symptoms suggestive of pre-eclampsia, whereas those who additionally reported experiencing convulsions (not from fever) were coded as eclamptic. However, it was not possible to confirm clinical diagnosis of these symptoms. Data on blood pressure and proteinuria during pregnancy, which are typical clinical diagnostic markers of pre-eclampsia [41], were not available in the NFHS-3. Data on physician reported diagnosis of convulsions/seizures were also not available in the NFHS-3 to verify a self-reported diagnosis.

### Key Predictors

Dietary diversity is often used as a proxy for dietary intake, both because it is a straightforward measure of nutrition, and also because the burden on respondents is relatively low [30]. The WHO [42] has identified 8 broadly defined food groups (grains, roots and tubers; legumes and nuts; dairy products; flesh foods; eggs; vitamin A-rich fruits and vegetables; and other fruits and vegetables), and suggests that individuals should eat from at least four food groups daily in order to achieve an adequately diversified dietary intake. Based on WHO criteria [42], we created a dietary diversity score from women’s self-reported frequency (daily, weekly, occasionally, or never) of their consumption of milk or curd, green leafy vegetables, other vegetables, fruits, pulses and beans, eggs, fish, and chicken or meat. For each food category, consumption of at least one food item from the category is worth 1 point; however, consumption of foods that fall into multiple categories (such as eggs, which are categorized both as flesh foods and eggs) is worth 2 points. A minimum of 4 points is necessary for an adequately diversified dietary intake [42]. However, as the NFHS-3 data do not contain consumption data for some of the WHO-defined categories (e.g. grains, roots and tubers), we have modified the score so that a dietary diversity score greater than or equal to three was considered to be an adequately diversified dietary intake, and less than three was considered inadequate. Dietary diversity at the time of the survey was taken as a proxy measure for dietary diversity during pregnancy.

The other main exposure variable used in our study was self-reported consumption of iron and folic acid supplementation during pregnancy. For the most recent birth, NFHS-3 asked to mothers “Were you given or did you buy any iron and folic acid tablets or syrup?” In addition, the duration (in days) of iron and folic acid supplementation was recorded, and was categorized based on proposed national guidelines and WHO guidelines [43]. As iron and folic acid supplementation were included in the same question rather than as separate survey items, we were not able to include them as independent predictors in our models. We created a dichotomous indicator of iron and folic acid supplementation.

### Control Variables

Given the unknown etiology of PE or E, it is possible that its onset is influenced by sociodemographic and health-related factors. In order to adjust for this, we controlled for three groups of potential confounders.

#### Maternal Factors

Categorical measures of age (15–29, 30–39, and 40–49 years) and birth interval (first child, <2 years, 2–3 years, 3 years or more) and a dichotomous indicator of pregnancy type (single, multiple) were included to control for the positive association between maternal age [44–45], short birth intervals, multiple births, and health risks during pregnancy. As pre-eclampsia and eclampsia risk has been found to be higher in nulliparous women [28, 46], we included a categorical measure of parity (1, 2, 3, or 4 or more). To control for the possibility that progression from gestational hypertension to pre-eclampsia may be increased by a prior miscarriage [47], we created a dichotomous indicator of whether the respondent ever had an induced or spontaneous abortion. As anemia during pregnancy has been linked to pre-eclampsia [48], we followed CDC recommendations [49] to construct a categorical measure of anemia level (not anemic, mildly anemic, moderately anemic, and severely anemic) adjusted for current pregnancy status, living at high altitudes, and smoking behaviour. In India, more than 80% of the pregnant women received iron and folic acid supplementation through antenatal care (ANC), and women were provided with information on proper nutrition and the signs and symptoms of pre-eclampsia and eclampsia during their ANC visit (38). Given the importance of appropriate ANC for maternal and child health, we thus included two controls for self-reported utilization of ANC: a categorical measure of the number of visits (none, 1–3, and 4 or more) and access to healthcare, measured by a categorical indicator of type of healthcare facility used (public medical sector, NGO trust hospital or clinic, private medical sector, and other sources).

#### Health and Lifestyle Factors

As dietary patterns are likely to be part of a broader health lifestyle, which may itself have implications for PE or E risk, we included a group of covariates to adjust for health and lifestyle factors. Studies have demonstrated that women with pre-eclampsia have a higher rate of pre-gestational hypertension and overweight or obese body mass index (BMI) [50]. Following this, we included a Asian Population standard [51–53] BMI (kg/m^2^) measure, categorized as underweight (≤18.5 kg/m^2^), normal (18.5 to 22.9 kg/m^2^), overweight (23.0 to 24.9 kg/m^2^), and obese (≥25.0 kg/m^2^). As change in BMI between pregnancies has been estimated to be over one-half a unit (median 0.7; IQR −0.3–1.7) [54], we assumed that there is unlikely to be a marked change in category of BMI between the birth and the time of the survey. We therefore used BMI at the time of the survey as a proxy for pre-pregnancy BMI. While maternal cigarette smoking increases the risk of a number of pregnancy complications, smoking has consistently been shown to reduce the risk of pre-eclampsia by approximately 30% in western studies [55–59]. In NFHS-3, participants were asked four yes/no questions on current use of cigarettes, pipes, other local tobacco smoking products, and snuff, chew, or other smokeless tobacco products. As a dichotomous measure of current tobacco use, we classified women as smokers if the response was ‘yes’ to smoking cigarettes, pipes, or other local smoking products. Women were classified as ‘smokeless tobacco users’ if the response was ‘yes’ to the use of chew, snuff, or other local smokeless tobacco products currently. As several studies have found a negative association between prenatal alcohol consumption and pre-eclampsia [60–62], we constructed a dichotomous indicator of current alcohol use. Due to the association between diabetes and maternal health, we also included dichotomous indicators of self-reported diabetes. Several studies have shown that women with gestational diabetes mellitus have an increased risk of pre-eclampsia or gestational hypertension [63–64] and that insulin resistance predates the development of pre-eclampsia, implying that insulin resistance may play a role in its etiology [65–66]. The risk of pre-eclampsia also increases with increasing glucose intolerance [64,67–68]. Some studies also suggest that asthmatics, particularly those who are symptomatic during pregnancy, may be at a higher risk of developing pre-eclampsia [69]. We thus included a dichotomous indicator of self-reported asthma in the current analysis.

#### Sociodemographic Characteristics

In order to reduce the risk of unobserved homogeneity in our models, we included a variety of socio-demographic controls. Religion was categorized into Hindu, Muslim, Christian, Sikhs or other/missing (Buddhist, Jain, Jewish, Zoroastrian etc.). Caste was categorized as scheduled caste, scheduled tribe, other backward class (NFHS-3 classification; hereafter referred to as disadvantaged class), general, and other/missing [38]. We also included categorical indicators of maternal education (no education, primary, secondary, and higher) and occupation (professional/ technical/managerial, clerical/sales, agricultural work, services, skilled and unskilled manual labour, and not working). Household wealth was categorized into quintiles based on the standard DHS index of household assets [37–38]. Place of residence was defined as either urban or rural. To account for differences in dietary preferences, food security subsidies, and other factors which may vary regionally, geographic regions was classified into six zones: north, northeast, central, east, west and south.

### Statistical Analysis

All analyses were conducted using the SPSS statistical software package Version 19 (IBM SPSS Statistics, Chicago, Illinois, USA). Standard descriptive statistics were calculated for all variables, including means and standard deviations. Differences in categorical variables were tested using χ^2^ tests. We assessed the possibility of multicollinearity between the covariates by using a correlation matrix of covariates. All pairwise Pearson correlation coefficients were less than 0.5, suggesting that multicollinearity is not a concern. We included NFHS-3 sampling weights to account for the sample design and ensure the representativeness of the sample [38]. Fixed effects multivariable logistic regression models [70–71] were then used to estimate the association between dietary diversity, iron and folic acid supplementation during pregnancy, and PE or E.

Multivariable models were adjusted for the above described maternal factors, health and lifestyle factors and socio-demographic characteristics of the mother. In the first logistic regression model, we examined the unadjusted association between dietary diversity, iron and folic acid supplementation and PE or E symptoms independent of each other. In the second model, we adjusted for maternal factors in order to assess how much of the variance in this association was explained by maternal characteristics. In the third model, we added health lifestyle factors to our model. In the fourth and final model, we added socio-demographic characteristics in order to examine the association between dietary diversity, iron and folic acid supplementation and PE or E symptoms controlling for all the confounders discussed above. Maternal marital status was not included as part of the socio-demographic characteristics because the majority (i.e., 98%) were married. We also tested for a variety of potential interactive effects, for any possible significant interactions. In particular, given that ANC visits may improve maternal health outcomes both by direct treatment of health conditions and by providing women with dietary advice and supplementation, we tested for an interaction between ANC visits with each of our two key predictors. State fixed effects were included in all models, and standard errors were clustered at the primary sampling unit.

It has been argued that logistic regression models should be used with a minimum of 10 events per predictor variable (EPV) [72–74]. In this etiological observational study, we are interested in adjusting for a number of covariates. Simulation studies show that 16–24 events per variable also produce stable effect estimates [75]. Given that our sample size is 39,657 participants, a conservative event rate of 50% is an acceptable ratio of events to non-events to adjust for the large number of covariates [75].

### Sensitivity analyses

Women with better socioeconomic resources may a) have a better understanding of their symptoms, b) report their experiences differently when compared to women in lower wealth and education groups, and c) have access to a better diet during pregnancy. To overcome potential sources of information bias, particularly with regard to self-reported diet and health problems during pregnancy, we ran sensitivity analyses for level of education (higher than secondary) and household wealth (richest quintile). Given that the population of women attending ANC might be inherently different than those who do not, we also conducted a sensitivity analysis on ANC visits, restricting the sample to only those women who attended ANC.

Women who were currently pregnant or who had given birth within 2 months of the survey were not weighed, resulting in missing values on the BMI measure for currently or recently pregnant women. Due to list wise deletion, these women were excluded from our full regression models. Given that pregnancy should in theory be randomly distributed in the sample, it is unlikely that this sample restriction would bias our sample. However, to test for potential bias, we created a variable to flag cases that were missing on BMI, and ran our fully adjusted model including the flag variable, but without BMI as a covariate in order to retain these cases. However, since BMI is an important covariate that could influence the effect estimate, we have again re-estimated the models by adding an additional category to the existing BMI variable to signify those who were missing on the measure.

We also repeated the analysis, restricting it to those women who had given birth up to 3 years prior to the survey and reported PE or E symptoms to examine the potential effects of recall bias. Short birth intervals are associated with adverse perinatal outcomes [76], but long intervals, especially those longer than 5 years, are also independently associated with an increased risk of pre-eclampsia [77]. In a final set of sensitivity analyses, we restricted the models to multiparous women (74% of the sample) who were not currently pregnant, and examined the effect of the length of the birth interval and PE or E risk.

## Results

Table 1 presents the characteristics of the study sample. Overall, 1.2% (n = 456) of mothers reported symptoms suggestive of PE or E. One-third of the mothers (31.1%) consumed an adequately diversified diet and one-fourth reported of taking iron and folic acid supplementation for at least 90 days during their last pregnancy, though almost two-thirds (65%) reported taking an iron and folic acid supplement at some point during their pregnancy. One-fourth of the mothers were at first parity, and 31% of the births were preceded by an interval of more than 3 years and 1% of the pregnancies were multiple births.

**Table 1:** Characteristics of the study participants (n = 39,657), India 2005–06.

Selected characteristics	Women age 15–49 years	
	Number	%
*Maternal factors*		
Pre-eclampsia or Eclampsia symptoms		
No	39155	98.8
Yes	456	1.2
Dietary diversity		
Inadequate	27275	68.9
Adequate	12337	31.1
Iron and folic acid supplementation		
No	13646	34.6
Yes	25809	65.4
Missing cases	157	0.4
Duration of iron and folic acid supplementation		
<90 days	29588	74.7
≥90 days	10024	25.3
Parity		
1	10453	26.4
2	11370	28.7
3	6810	17.2
4 and above	11005	27.8
Preceding birth interval		
First order birth	10546	26.6
Interval <2 years	7124	18.0
Interval 2-3 years	9538	24.1
Interval 3 years or more	12448	31.4
Type of pregnancy		
Singleton	39298	99.1
Multiple	359	0.9
Terminated pregnancy		
Never	32319	81.5
Ever	7338	18.5
Anaemia level		
Not anaemic	14939	40.1
Mild	15082	40.4
Moderate	6616	17.7
Severe	652	1.7
Missing cases	2362	6.0
Number of ANC visits		
No visit	9020	22.8
1-3	9696	24.5
4 or more	20895	52.8
Healthcare access		
Public medical sector	11303	31.3
NGO Trust Hospital/Clinic	113	0.3
Private Medical Sector	24591	68.1
Other sources	119	0.3
Missing cases	3486	8.8
*Health and Lifestyle factors*		
Body Mass Index		
Underweight (≤18.5 kg/m^2^)	11592	30.5
Normal (18.5-22.9 kg/m^2^)	20714	54.4
Overweight (23.0-24.9 kg/m^2^)	2770	7.3
Obese (≥25.0 kg/m^2^)	3226	14.7
Missing cases	1609	4.1
Smoking tobacco		
No	39049	98.5
Yes	608	1.5
Smokeless tobacco use		
No	36333	91.7
Yes	3278	8.3
Drinks alcohol		
No	38735	97.7
Yes	911	2.3
Missing cases	11	0.0
*Current medical conditions*		
Diabetes		
No	39123	98.7
Yes	160	1.3
Missing cases	20	0.1
Asthma		
No	39163	98.8
Yes	470	1.2
Missing cases	24	0.1
*Socioeconomic and demographic factors*		
Age		
15-29 y	29190	73.6
30-39 y	9421	23.8
40-49 y	1047	2.6
Education		
No education	18783	47.4
Primary	5550	14.0
Secondary	12959	32.7
Higher	2365	6.0
Missing cases	1	0.0
Religion		
Hindu	31280	78.9
Muslim	6482	16.3
Christian	814	2.1
Sikhs	514	1.3
Others	568	1.4
Caste/tribe		
Scheduled caste	7945	20.1
Scheduled tribes	3742	9.5
Disadvantaged class	15878	40.2
General	10845	27.5
Missing caste	1089	2.8
Missing cases	158	0.4
Occupation		
Not working	24710	62.4
Professional/technical/managerial	670	1.7
Clerical/Sales	566	1.4
Agricultural work	9950	25.1
Services	715	1.8
Skilled and unskilled-Mannual	2991	7.6
Missing cases	9	0.0
Wealth index		
Poorest	9566	24.1
Poorer	8600	21.7
Middle	7769	19.6
Richer	7256	18.3
Richest	6466	16.3
Place of residence		
Urban	10622	26.8
Rural	29035	73.2
Geographic Regions		
North	5678	12.8
Northeast	1613	4.1
Central	11111	28.0
East	10042	25.3
West	5117	12.9
South	6696	16.9
Total	39657	100.0

Note: Number of women varies slightly for individual variables depending on the number of missing values

Approximately one-fifth (18.5%) of the mothers reported a spontaneous or induced abortion. Malnutrition rates were high: almost one-fifth of the mothers were moderately or severely anaemic, 31% were underweight while 22% were either overweight or obese. More than half the mothers had four or more ANC visits during their last pregnancy and almost 70% had access to the private medical sector to obtain their health care. Very few were current smokers (1.5%) or alcohol drinkers (2.3%), while almost one in ten were smokeless tobacco users (8.3%). Prevalence of diabetes (1.3%) and asthma (1.2%) were low. Most mothers (almost three-fourth) were aged 15–29 years, and almost half (47.4%) had no education. A majority of the mothers (four out of five) were identified as Hindu, and two-fifths belonged to a disadvantaged class. One in four mothers were agricultural worker while a very few (1.8%) worked in the service sector. One fourth belonged to the household with poorest wealth. More than 70% of the mothers were residing in rural areas and 28% were residents of Central India.

Table 2 provides the percentage distribution of PE or E cases and prevalence of symptoms of PE or E according to selected characteristics. Of women reporting PE or E symptoms, more than four-fifths reported of inadequately diversified dietary intake; half had consumed iron and folic acid supplementation and 13% adhered to the recommended duration (≥90 days) of IFA intake; two-fifths (41%) were four and higher order births; 39% had a birth interval of 3 years or more; 2.4% were multiple pregnancies; one-fourth had terminated pregnancies; almost half (46%) were mildly anemic; more than two-thirds (71%) had access to public medical sector for healthcare needs; 3% were obese; 2% was smoking tobacco; 13% were smokeless tobacco users; 5% drinks alcohol; 1.8% and 5% reported having diabetes or asthma respectively; two-thirds were in the age group 15–29 years; two-thirds had no education; 80% were Hindus; 38% belonged to disadvantaged class; half were not working; two-fifths belong to poorest wealth quintile; a majority resides in rural area whereas two-fifths resides in central India.

**Table 2:** Percentage distribution of Pre-eclampsia or eclampsia cases and self-reported prevalence of symptoms suggestive of Pre-eclampsia or eclampsia according to selected characteristics, India 2005–06.

Variables	Total Number of Pre-eclampsia or Eclampsia cases	% Distribution of Pre-eclampsia or Eclampsia cases	% Prevalence of Pre-eclampsia or Eclampsia	Χ^2^ p value
*Diet and iron and folic acid intake*				<0.0001
Dietary diversity				
In adequate	377	82.5	1.4	
Adequate	80	17.5	0.6	
Iron and folic acid supplementation				<0.0001
No	217	47.7	1.6	
Yes	238	52.3	0.9	
Duration of iron and folic acid supplementation				<0.0001
<90 days	398	87.3	1.3	
≥90 days	58	12.7	0.6	
*Maternal factors*				
Parity				<0.0001
1	85	18.6	0.8	
2	103	22.6	0.9	
3	81	17.8	1.2	
4 and above	187	41.0	1.7	
Preceding birth interval				<0.0001
First order birth	85	18.6	0.8	
Interval <2 years	88	19.3	1.2	
Interval 2-3 years	104	22.8	1.1	
Interval 3 years or more	179	39.3	1.4	
Type of pregnancy				0.003
Singleton	446	97.6	1.1	
Multiple	11	2.4	3.1	
Terminated pregnancy				0.005
Never	350	76.8	1.1	
Ever	106	23.2	1.4	
Anemia level				<0.0001
Not anaemic	129	29.6	0.9	
Mild	200	45.9	1.3	
Moderate	101	23.2	1.5	
Severe	6	1.4	0.9	
Number of ANC visits				<0.0001
No visit	149	32.6	1.7	
1-3	148	32.4	1.5	
4 or more	160	35.0	0.8	
Healthcare access				0.190
Public medical sector	124	28.8	1.1	
NGO Trust Hospital/Clinic	3	0.7	2.7	
Private Medical Sector	304	70.5	1.2	
Other sources	0	0.0	0.0	
*Health and Lifestyle factors*				
Body Mass Index				0.001
Underweight (≤18.5 kg/m^2^)	183	50.3	1.3	
Normal (18.5-22.9 kg/m^2^)	224	41.1	1.3	
Overweight (23.0-24.9 kg/m^2^)	23	5.2	0.8	
Obese (≥25.0 kg/m^2^)	15	3.4	0.5	
Smoking Tobacco				0.164
No	446	97.8	1.1	
Yes	10	2.2	1.7	
Smokeless tobacco use				<0.0001
No	397	87.1	1.1	
Yes	59	12.9	1.8	
Drinks Alcohol				<0.0001
No	433	94.7	1.1	
Yes	24	5.3	2.6	
*Current medical conditions*				
Diabetes				0.240
No	448	98.2	1.1	
Yes	8	1.8	1.6	
Asthma				<0.0001
No	434	95.2	1.1	
Yes	22	4.8	4.7	
*Socioeconomic and demographic factors*				
Age				0.013
15-29 y	309	67.6	1.1	
30-39 y	134	29.3	1.4	
40-49 y	14	3.1	1.3	
Education				<0.0001
No education	294	64.5	1.6	
Primary	66	14.5	1.2	
Secondary	91	20.0	0.7	
Higher	5	1.1	0.2	
Religion				0.027
Hindu	362	79.6	1.2	
Muslim	69	15.2	1.1	
Christian	6	1.3	0.7	
Sikhs	4	0.9	0.8	
Others	14	3.1	2.5	
Caste/tribe				<0.0001
Scheduled caste	99	21.8	1.2	
Scheduled tribes	84	18.5	2.2	
Disadvantaged class	173	38.0	1.1	
General	88	19.3	0.8	
Missing caste	11	2.4	1.0	
Occupation				<0.0001
Not working	228	50.0	0.9	
Professional/technical/managerial	8	1.8	1.2	
Clerical/Sales	3	0.7	0.5	
Agricultural work	173	37.9	1.7	
Services	71	1.5	1.0	
Skilled and unskilled-Manual	37	8.1	1.2	
Wealth index				<0.0001
Poorest	187	41.1	2.0	
Poorer	109	24.0	1.3	
Middle	76	16.7	1.0	
Richer	47	10.3	0.6	
Richest	36	7.9	0.6	
Place of residence				<0.0001
Urban	65	14.2	0.6	
Rural	392	85.8	1.4	
Geographic Regions				<0.0001
North	52	11.4	1.0	
Northeast	22	4.8	1.4	
Central	188	41.2	1.7	
East	137	30.0	1.4	
West	36	7.9	0.7	
South	21	4.6	0.3	
Total	456	100.0	1.2	

Considering prevalence of PE or E, a higher proportion of women with inadequate dietary diversity reported of PE or E symptoms (1.4%) than those women with adequately diversified dietary intake (0.6%). A higher proportion of those who did not consume iron and folic acid supplementation for at least 90 days also reported of high PE or E (1.3%) symptoms as compared to those who took iron and folic acid supplements for 90 or more days (0.6%). Women with mild (1.3%) and moderate anaemia (1.5%) also reported higher symptoms of PE or E as compared to those with no (0.9%) or severe (0.9%) anaemia. Compared to overweight (0.8%) or obese (0.5%) women, those who were underweight (1.3%) and normal weight (1.3%) reported higher proportion of PE or E symptoms.

Pregnancy and health risk factors were also significantly associated with PE or E symptoms, with a higher proportion of women of parity 4 or higher (1.7%) reported higher PE or E symptoms than women of lower parity. First order births had the lowest proportion of PE or E symptoms (0.8%), while women with a birth interval of 3 or more years experienced significantly higher symptoms of PE or E (1.4%). Multiple births were also associated with higher odds of PE or E symptoms (3.1%) compared to singleton births (1.1%), as were previously aborted pregnancies (1.4%) compared to those with no previous spontaneous or induced abortions (1.1%). A higher proportion of current smokeless tobacco users (1.8%), current alcohol drinkers (2.6%), and current tobacco smokers (1.7%) also reported PE or E symptoms compared to those who, respectively, do not currently use tobacco, drink, or smoke. Additionally, asthmatics (4.7%) reported higher PE or E symptoms than non-asthmatics (1.1%).

Significant associations were also found between socio-demographic characteristics and PE or E symptoms. A lower proportion of women aged 15–29 reported PE or E symptoms (1.1%) compared to women aged 30–39 (1.4%) and 40–49 years (1.3%). However, a higher proportion of women with no education (1.6%) reported PE or E symptoms compared to those with primary, secondary, or higher levels of education. Caste was also a significant correlate, with a higher proportion of women reporting PE or E symptoms belonging to a scheduled tribe (2.2%) than those in a scheduled caste (1.2%), disadvantaged class (1.1%) or general caste (0.8%). Those in the poorest wealth quintile reported the highest PE or E symptoms (2.0%) compared to those in the other four wealth quintiles, as did those in professional (1.2%) and agricultural (1.7%) occupations. Rural residence was also associated with increased proportion of women reporting a PE or E symptoms (1.4%) compared to urban residence (0.6%), and women living in central (1.7%), northeast (1.4%) and eastern (1.4%) India reported higher PE or E symptoms than those in other regions.

Table 3 shows results of multivariable logistic regression analyses of the association between dietary diversity, iron and folic acid supplementation during pregnancy, and symptoms suggestive of PE or E, in unadjusted, partially adjusted and fully adjusted models. In the unadjusted analysis (Model 1), the likelihood of reporting PE or E symptoms was significantly lower among women who had an adequately diversified dietary intake (OR: 0.52; 95% CI: 0.41–0.66) than among those who did not have a diversified dietary intake. Similarly, women who took iron and folic acid supplements for 90 or more days during pregnancy had a lower likelihood of reporting PE or E symptoms (OR: 0.48; 95% CI: 0.36–0.64) than those who did not take iron or folic acid supplements for the recommended 90 days. Controlling for the maternal factors (in Model 2) slightly attenuated the inverse relationship between diversified dietary intake (OR: 0.57; 95% CI: 0.44–0.73) and PE or E, as well as between intake of iron and folic acid supplementation (OR: 0.61; 95% CI: 0.45–0.8) and PE or E symptoms. The inverse association between the effect of diversified dietary intake (OR: 0.56; 95% CI: 0.43–0.72), intake of iron and folic acid supplementation (OR: 0.56; 95% CI: 0.42–0.75) and PE or E symptoms remained virtually unchanged when health and lifestyle factors were additionally controlled for in Model 3. The final model (Model 4) in Table 3 provides the fully adjusted model with maternal factors, health and lifestyle factors, and socio-demographic characteristics included. Of the significant predictors from the bi-variable associations, twin pregnancy, ever having an abortion, mild or moderate anaemia, being asthmatic, occupation, wealth quintile, and caste remained significant predictors of PE or E symptoms in the fully adjusted model (Model 4), while parity, birth interval, BMI, alcohol and tobacco use, age, education, place of residence, and geographic region were no longer significant. Jointly controlling for all of these factors, the inverse association between an adequately diversified dietary intake (OR: 0.66; 95% CI: 0.51–0.87), recommended intake of iron and folic acid supplementation (OR: 0.64; 95% CI: 0.47–0.88) and PE or E symptoms during pregnancy remains strong and statistically significant. We found no evidence of an interaction effect between dietary diversity, iron and folic acid supplementation and ANC utilization in the fully adjusted model.

**Table 3:** Unadjusted and adjusted odds ratios (ORs) and 95% confidence interval (95%CI) showing the association between adequately diversified dietary intake, iron and folic acid supplementation and symptoms of pre-eclampsia or eclampsia,India, 2005–06.

Key predictors	Model 1 Unadjusted	Model 2 Adjusted	Model 3 Adjusted	Model 4 Adjusted
	OR[95%CI]	OR[95%CI]	OR[95%CI]	OR[95%CI]
*Diet and iron and folic acid intake*				
Dietary diversity				
Adequate	0.52[0.41–0.66]	0.57[0.44–0.73]	0.56[0.43–0.72]	0.66[0.51–0.87]
Inadequate ^R^	1.00	1.00	1.00	1.00
P value	<0.0001	<0.0001	<0.0001	0.003
Duration of iron and folic acid supplementation				
≥90 days	0.48[0.36–0.64]	0.61[0.45–0.82]	0.56[0.42–0.75]	0.64[0.47–0.88]
<90 days ^R^	1.00	1.00	1.00	1.00
P value	<0.0001	0.001	<0.0001	0.006

^R^ Indicates reference category; Sample size is 39,657

Model 1 unadjusted

Model 2 adjusted for maternal factors only

Model 3 adjusted for maternal factors, health and lifestyle factors and current medical conditions

Model 4 adjusted for maternal, health and lifestyle factors, current medical conditions and socio-demographic characteristics

### Results of the sensitivity analysis

As a robustness check, we ran Models 1 through 4 restricting only to those cases with valid data on the BMI measure. Results remained largely unchanged: diversified dietary intake (OR: 0.67; 95% CI: 0.54–0.85; p = 0.004) and intake of iron and folic acid supplementation (OR: 0.65; 95% CI: 0.48–0.89; p = 0.006) continued to be significant predictors of lower occurrence of PE or E symptoms in Indian women (*not shown on the table*). We again re-estimated the models with BMI split into 5 categories (underweight, normal, overweight, obese, and missing) and could found no demonstrable change in the association. Among women in the highest education group, the likelihood of reporting symptoms of PE or E was lower among those with a adequately diversified dietary intake (OR: 0.60; 95% CI: 0.39–0.78) and recommended intake of iron and folic acid supplementation (OR: 0.68; 95% CI: 0.45–0.98) during pregnancy (*not shown on the table*). Even after additional adjustment for ANC visits, intake of an adequately diversified diet was associated with a 43% (OR: 0.57; 95% CI: 0.44–0.73) and iron and folic acid supplementation was associated with a 41% reduction in the likelihood of PE or E (OR: 0.59; 95% CI: 0.49–0.80) symptoms. Restricting our analysis to those women who had given birth up to 3 years prior to the survey and reported PE or E symptoms (1.4%), we found that the association of adequately diversified dietary intake, iron and folic acid supplementation, and PE or E symptoms was non-significant. However, the number of women in this restricted sample was very small (n = 46), and as a result the models had limited statistical power. In a final set of sensitivity analyses restricting the models to multiparous women we found that the significant inverse association between diversified dietary intake, intake of iron and folic acid supplementation during pregnancy and symptoms of PE or E still remained.

## Discussion

In this study, we examined the association between adequately diversified dietary intake, iron and folic acid supplementation during pregnancy, and symptoms suggestive of PE or E in a large, nationally representative sample of Indian women. Our main analyses provide empirical evidence of an inverse association between an adequately diversified dietary intake, iron and folic acid supplementation during pregnancy and occurrence of symptoms suggestive of PE or E. This association remained significant after controlling for a wide range of socio-demographic, maternal, and health and lifestyle factors. Overall 1.2% of the study sample experienced symptoms suggestive of PE or E in their most recent pregnancy. Mothers who had a adequately diversified dietary intake during pregnancy were 34% less likely to report PE or E symptoms than mothers with inadequately diversified dietary intake. The likelihood of reporting PE or E symptoms was also 36% lower among those mothers who consumed iron and folic supplementation for at least 90 days during their last pregnancy. Results from the sensitivity analyses suggests that among highly educated mothers, the likelihood of reporting symptoms of PE or E was 40% lower among those with an adequately diversified dietary intake and recommended intake of iron and folic acid supplementation during pregnancy. Even after additional adjustment for ANC visits, intake of an adequately diversified diet and recommended intake of iron and folic acid supplementation were associated with a significantly lower likelihood of reporting PE or E symptoms. Restricting our analysis to only multiparous women we found that the significant, inverse association between diversified dietary intake, recommended intake of iron and folic acid supplementation during pregnancy and symptoms of PE or E remained significant.

There is a mounting evidence that nutrition plays an important role in preventing hypertensive disorders [14–15, 78–81], and maternal nutritional status has long been hypothesized to have a role in the pathophysiology of PE or E [10, 29, 80, 82–90], especially in developed countries. There is also a strong evidence from both animal and human studies to support the hypothesized protective effect of micronutrients such as iron and folic acid on low PE or E risk [12, 15, 17–19]. Our study supports previous research [91–97] by providing population-level evidence from a developing country that iron and folic acid supplementation may reduce the symptoms of PE or E in pregnancy. Use of the National Family Health Survey data also provided adequate sample size to study the dietary correlates of PE or E in a population-level study, thus providing a unique opportunity for descriptive inferences about the social distribution and patterning of PE or E risk among Indian women.

### Limitations of the study

There are several limitations to our study. First, most variables in the analyses (with the exception of anthropometrics) were self-reported, including a symptomatic rather than clinical measure of PE or E; it is possible that self-reported data may suffer from recall bias. Although we cannot rule out the possibility of misclassification within this context, it is unlikely that we have missed severe PE or E cases due to the generally clear manifestation of symptoms in severe cases. Second, due to the nature of the data, we could not identify the gestational onset of PE or E; it is possible that nutritional factors may be particularly important during critical periods of foetal development, which we are not able to assess in this study. Third, the role of folic acid independent of iron supplementation may be of interest. However, because iron and folic acid were measured jointly rather than as separate supplements in NFHS-3, we were unable to disentangle the effects of these two supplements. Similarly, information about other micronutrient supplementation is unavailable in the NFHS-3; while our models control for a wide range of health and sociodemographic factors, it is possible that other micronutrients, such as B12, may also shape PE or E risk. Further research is needed at the population level to understand the independent effects of micronutrient supplementation. An additional concern regarding the self-reported data is potential misreporting of dietary intake; this misreporting would most likely be non-differential, and may attenuate the association between dietary diversity and PE or E symptoms. Moreover, the broad categories of food intake in the NFHS-3 limits our ability to assess the impact of particular food items, especially those associated with diabetes (such as sugar) and other health conditions. An adequately diversified dietary intake was associated with higher socioeconomic status in our study; higher socioeconomic status may be associated with both better diets and regular health check-ups. However, the results of our sensitivity analyses of models stratified by wealth, education, and ANC visits suggest that this bias is limited.

In our sample, the prevalence of PE or E symptoms was quite low (1.2%) as compared to other studies in India. In part, this low prevalence is likely reflects the fact that survey participants were selected from the general population rather than from the higher-risk populations used in many clinical and epidemiological studies. Additionally, because we were unable to confirm PE or E with a clinical diagnosis, we have used a particularly stringent measure, requiring women to have experienced *both* difficulties with daytime vision and swelling of the face and extremities during pregnancy. This more stringent definition may result in a conservative bias in our results, as some women who reported experiencing only one symptom (and thus were coded as not experiencing PE or E here) may have been identified as pre-eclamptic via clinical diagnosis.

Our study also showed the prevalence of PE or E was higher among women of higher parity, lean women, current smokers, and the less-educated. While these findings are in contrast to some of the existing literature conducted in developed countries, they are in keeping with other work among Asian women [46,98–99]. Our fully adjusted multivariable analyses results also fit with previous literature pointing to increased risk of PE or E symptoms for: multiple (rather than in singleton) pregnancies [100–102]; current smokers [55–56,103]; mothers with a history of spontaneous or induced abortion [104–105]; women with asthma [106–107] and diabetes [63,108]; and those from disadvantaged socio-demographic groups [47,109]. Lower age and parity were not found to be associated with pre-eclampsia in contrast to other studies [110–111]. The disparity between our study and other studies could be due to the differences in the population-based sample here compared to hospital-based samples elsewhere. Our finding that current tobacco smoking is associated with significantly increased risk of pre-eclampsia is also consistent with previous research [55,112] though an earlier systematic review analyzing the pooled data from cohort and case-control studies showed a lower risk of pre-eclampsia associated with cigarette smoking during pregnancy [56,103]. However, information on smoking and alcohol here referred to the current lifestyle of the respondent rather than behaviour during pregnancy, which may explain why our study departs from previous findings. Our findings concur with previous studies regarding risks of PE or E arising from medical conditions such as asthma, ethnicity [113], socio-economic position [109], and history of miscarriage or terminated pregnancy [104–105]. However, a minor limitation of the present study is the inability to differentiate between spontaneous and induced abortion because of the lack of information on this variable.

Adverse maternal and infant health outcomes often can be prevented by modifying maternal behaviours [35]. Monitoring behavioural risk factors can provide direction for interventions to improve the health of mothers and infants. The current study extends previous research by assessing the role of dietary factors and intake of iron and folic acid supplementation in pregnancy in shaping the PE or E risk. Low or inadequate diversified diet is particular problematic in low- and middle-income countries (LMICs) [114–115], where the diets contain a disproportionate share of starchy staples (e.g. rice, maize, potatoes) and wheat, while intake of animal-source foods (meat, fish, eggs and dairy) and fresh fruit and vegetables is often insufficient. In addition, certain staple foods, such as wheat, maize, and millet, can contain high levels of anti-nutrients (e.g. phytates), which reduce the absorption of available micronutrients [115]. Many of the important vitamins and minerals essential for a healthy diet are found in greatest abundance in fruits and vegetables, legumes, and flesh foods. The recent dietary transition in developing countries [116] towards low-cost processed grains, oils, and sugar has led to a reduced intake of micronutrients and a progressive loss of dietary diversity. As a large proportion of Indian women subsist on iron-poor vegetarian diets [117], large-scale iron supplementation and fortification of commonly consumed vegetarian foodstuffs constitutes a feasible, culturally appropriate, and cost-effective strategy for reducing pregnancy induced PE or E symptoms. However, iron and folic acid supplementation needs to be placed in the broader context of maternal nutritional status in India.

With the target of the Millennium Development Goals and adherence to India’s Five Year Plan for reproductive health in sight, PE or E should be identified as a priority area in reducing maternal morbidity in India. Dietary diversity and nutritional adequacy in pregnancy represent modifiable risk factors for PE or E, and should be considered as targets for prevention. Efforts which exclusively target micronutrient supplementation may not be sufficient for reducing PE or E symptoms. Policies aiming to reduce the risk of hypertension in pregnancy should target improving access to, and compliance with, the simple recommended therapy of iron and folic acid supplementation and an adequately diversified dietary intake in tandem. However, as this analysis has demonstrated, there may be a substantial socioeconomic gradient in health risk behaviour and access to preventive care. Programmatic interventions should focus particularly on women with limited socioeconomic resources. For example, programs targeting food security among low income groups in India, such as the Public Distribution System (PDS), could improve maternal and child health and reduce the risk of PE or E by offering a more diversified basket of subsidized food items.

## Conclusions

Using nationally representative data on reproductive health among Indian women, our study shows that having an adequately diversified dietary intake and iron and folic acid supplementation in pregnancy is associated with a reduced occurrence of symptoms suggestive of PE or E in Indian women. This relationship persists when sociodemographic, health lifestyle, and maternal factors are taken into consideration. Because ANC is the main source of iron and folic acid supplements and nutrition education during pregnancy, ensuring universal provision of comprehensive ANC, emphasized in India’s Eleventh Five Year Plan (2007–2012) [118], is vital for improving maternal nutrition and health in India. Further epidemiological research in India with large-scale cohorts should focus on uncovering preventable causes of PE or E such as diet and micronutrient supplementation during pregnancy, while public health practice and policy should promote improved access to better food and nutrition and mandatory intake of iron and folic acid supplementation during pregnancy among Indian mothers.
